# Improved Progression-Free Survival in Irinotecan-Treated Metastatic Colorectal Cancer Patients Carrying the HNF1A Coding Variant p.I27L

**DOI:** 10.3389/fphar.2017.00712

**Published:** 2017-10-10

**Authors:** Adrien Labriet, Elena De Mattia, Erika Cecchin, Éric Lévesque, Derek Jonker, Félix Couture, Angela Buonadonna, Mario D’Andrea, Lyne Villeneuve, Giuseppe Toffoli, Chantal Guillemette

**Affiliations:** ^1^Pharmacogenomics Laboratory, Centre Hospitalier Universitaire (CHU) de Québec Research Center, Québec, QC, Canada; ^2^Faculty of Pharmacy, Laval University, Québec, QC, Canada; ^3^Clinical and Experimental Pharmacology, IRCCS National Cancer Institute ‘Centro di Riferimento Oncologico’, Aviano, Italy; ^4^Centre Hospitalier Universitaire (CHU) de Québec Research Center, Québec, QC, Canada; ^5^Faculty of Medicine, Laval University, Québec, QC, Canada; ^6^Division of Medical Oncology, Department of Medicine, Ottawa Hospital, University of Ottawa, Ottawa, ON, Canada; ^7^Medical Oncology Unit, IRCCS National Cancer Institute ‘Centro di Riferimento Oncologico’, Aviano, Italy; ^8^Medical Oncology Unit, San Filippo Neri Hospital, Rome, Italy

**Keywords:** metastatic colorectal cancer, hepatocyte nuclear factor 1-alpha, irinotecan, polymorphism, progression-free survival

## Abstract

Hepatocyte nuclear factor 1-alpha (HNF1A) is a liver-enriched transcription factor that plays a key role in many aspects of hepatic functions including detoxification processes. We examined whether *HNF1A* polymorphisms are associated with clinical outcomes in two independent cohorts combining 417 European ancestry patients with metastatic colorectal cancer (mCRC) treated with irinotecan-based chemotherapy. The intronic rs2244608A>G marker was predictive of an improved progression-free survival with a trend in the Canadian cohort and reaching significance in the Italian cohort, with hazard ratios (HR) of 0.74 and 0.72, *P* = 0.076 and 0.038, respectively. A strong association between rs2244608A>G and improved PFS was found in the combined analysis of both cohorts (HR = 0.72; *P* = 0.002). Consistent with an altered HNF1A function, mCRC carriers of the *rs2244608G* minor allele displayed enhanced drug exposure by 45% (*P* = 0.032) compared to non-carriers. In Caucasians, rs2244608A>G is in strong linkage with the coding variant rs1169288c.79A>C (HNF1A p.I27L). In healthy donors, we observed an altered hepatic (*ABCC1*, *P* = 0.009, *ABCC2*, *P* = 0.048 and *CYP3A5*, *P* = 0.001; *n* = 89) and intestinal (*TOP1*, *P* = 0.004; *n* = 75) gene expression associated with the *rs1169288C* allele. In addition, the *rs1169288C* polymorphism could significantly increase the *ABCC1* promoter activity by 27% (*P* = 0.008) and 15% (*P* = 0.041) in the human kidney HEK293 and the human liver HepG2 cell lines, respectively. Our findings suggest that the *HNF1A* rs2244608, or the tightly linked functional coding variant p.I27L, might be a potential prognostic marker with irinotecan-based regimens.

## Introduction

The camptothecin derivative irinotecan (CPT-11) is a widely used chemotherapeutic agent for the treatment of solid tumors, particularly for metastatic carcinoma of the colon or rectum. A variety of first-line regimens such as irinotecan, 5-fluorouracil (5-FU) and leucovorin (FOLFIRI) used for mCRC in combination with targeted therapy improves outcomes (Fujita et al., 2015). Its active metabolite SN-38 inhibits the replication enzyme topoisomerase I (TOP1), inducing DNA damage and cell death in actively replicating cells leading to S-phase-specific cell killing. The clinical benefits from irinotecan-based regimens are variable among patients whereas sequencing and method of administration influence toxicity. Predictive and prognostic biomarkers are critical in identifying those who would most benefit from the chemotherapy. A number of genetic variants in genes involved in irinotecan pharmacology have been described and may help predict benefit of irinotecan-based chemotherapy. Most notably, the polymorphism *UGT1A1^∗^28*, leading to a decreased UGT1A1 enzyme expression and a reduced glucuronidation of the active metabolite SN-38, is a well-established marker of irinotecan-induced severe neutropenia and help guide dosing for treatment of mCRC (Beutler et al., 1998; Barbarino et al., 2014). In addition to help in decreasing the incidence of adverse drug reactions, pharmacogenetic markers can also be useful in predicting irinotecan responsiveness. Irinotecan pharmacology is complex and involves activation into SN-38 by carboxylesterases (CES), inactivation of SN-38 by UDP-glucuronosyltransferases (UGT)-dependent glucuronidation and cytochrome P450 (CYP)-dependent oxidation and hepatic transport of irinotecan and its metabolites by multiple transporters, all potentially contributing to the bioavailability and pharmacological activity of SN-38 (Smith et al., 2006).

Whereas genetic variants in genes regulating irinotecan pharmacology have been investigated, the role of nuclear receptors and other transcriptional factors has not been investigated, despite their potentially broader influence on drug metabolism (Cecchin et al., 2016; De Mattia et al., 2016). Hepatocyte nuclear factor 1-alpha (HNF1A) is a master hepatic transcriptional regulator that influences the expression of numerous genes critical to liver functions. Consistent with an important role in hepatic functions, *HNF1A* locus and genetic variations in *HNF1A* are associated with maturity-onset diabetes of the young (MODY3) (Yamagata et al., 1996), type 2 diabetes (Holmkvist et al., 2006; Voight et al., 2010), and coronary artery disease (Liu et al., 2014; Zhou et al., 2017). HNF1A also regulates a number of key genes involved in drug metabolism and disposition including *UGT* and ATP-binding cassette sub-family C member 2 (*ABCC2)* expression (Odom et al., 2004, 2007; Aleksunes et al., 2009; Qadri et al., 2009; Hu et al., 2014). Here, we hypothesized that polymorphisms in *HNF1A* may influence irinotecan pathways and predict clinical outcomes in mCRC patients. We tested the predictive significance of htSNPs in two independent cohorts of mCRC patients of European descent from Canada and Italy treated with the FOLFIRI regimen. We also studied the link with drug exposure in mCRC patients and tissular expression of genes potentially targeted by HNF1A and relevant to irinotecan pharmacology.

## Materials and Methods

### Patient Characteristics

A first cohort of 167 mCRC Canadian patients and a second cohort comprising 250 mCRC Italian patients were studied. Eligibility criteria included no prior irinotecan-based chemotherapy, histologically confirmed mCRC, a life expectancy of at least 3 months and a good performance status (Eastern Cooperative Oncology Group/World Health Organization Performance Status ≤ 2). White patients from the Canadian cohort were recruited from 2003 to 2012 in three medical centers of eastern Canada and those from the Italian cohort were enrolled from 2002 to 2005 in thirteen medical centers of Northeast Italy. All the patients from the Canadian cohort and the majority (>90%) of the Italian patients were treated with the modified FOLFIRI regimen (irinotecan 180 mg/m^2^ intravenously for 2 h on day 1 plus 5-fluorouracil (5-FU) 400 mg/m^2^ bolus followed by continuous infusion for 46 h of 5-FU 2400 mg/m^2^ plus leucovorin 200 mg/m^2^) every 2 weeks. The remaining Italian patients received a FOLFIRI regimen (irinotecan 180 mg/m^2^ intravenously for 2 h on day 1 plus 5-FU 400 mg/m^2^ bolus followed by 5-FU 600 mg/m^2^ continuous infusion during 22 h on days 1 and 2 + leucovorin 200 mg/m2 on days 1 and 2 every 2 weeks). Sixty-nine patients from the Canadian cohort received bevacizumab as a co-treatment and six others received another drug or a placebo. Genomic DNA isolation from blood samples was as described (Toffoli et al., 2006; Levesque et al., 2013). For a subset of Italian mCRC patients with available *HNF1A* genotype (*n* = 49), pharmacokinetics data including total plasma concentration of irinotecan and its metabolites, SN-38 and SN-38G assessed on serial blood samples collected after drug administration and using high-performance liquid chromatography, were available (Toffoli et al., 2006). Biliary index was calculated with the formula: [CPT-11 AUC X (SN-38 AUC/SN-38G AUC)] and glucuronidation ratio with the formula: (SN-38G AUC/SN-38 AUC). OS, PFS and response rate data in relation to patient characteristics and toxicity in both cohorts are shown in **Table 1**. This study was carried out in accordance with the regulatory framework for chemical and biological hazards of the CHU de Québec and Centro di Riferimento Oncologico di Aviano. All subjects provided written informed consent in accordance with the Declaration of Helsinki. The protocol was approved by the Comitato Etico Indipendente- Centro di Riferimento Oncologico di Aviano and the CHU de Québec ethics committees.

**Table 1:** Characteristics of the study cohorts of mCRC Caucasian patients treated with irinotecan-based chemotherapy (FOLFIRI regimens).

	Canadian cohort		Italian cohort	
		
Characteristics	*N* = 167	%	*N* = 250	%
Gender				
Male	110	66	162	65
Female	57	34	88	35
Age (years)				
Mean	61.5	–	60.6	–
Standard deviation	10.2	–	10.3	–
Range	29–86	–	26–75	–
FOLFIRI	167	100	250	100
Co-treatment	75	44.9	0	0
Bevacizumab	69	92.0	0	0
Other drug	6	8.0	0	0
Stage at diagnosis				
I	4	2.4	5	2.0
II	12	7.2	20	8.0
III	45	26.9	65	26.0
IV	100	59.9	160	64.0
Unknown	6	3.6	0	0
Outcome				
PFS (median in months)	11	–	7	–
OS (median in months)	24	–	15	–
Tumor response rate				
CR + PR	78	50.0	103	43.3
SD + PD	78	50.0	135	56.7
Severe toxicities (grades 3-4)				
Neutropenia	28	16.8	35	14.0
Diarrhea	24	14.4	21	8.4


*Stage at diagnosis evaluated by TNM system. CR, complete response; PR, partial response; SD, stable disease; PD, progressive disease; PFS, progression-free survival; OS, overall survival.*

### Genetic Analysis

Single-nucleotide polymorphisms in the *HNF1A* gene and 5 kb flanking regions were identified using the CEU population of the International HapMap Project information^1^. htSNPs were selected using Haploview v4.2 to tag for SNPs in high LD (*r*^2^ > 0.8) (Broad Institute, Cambridge, MA, United States) (Barrett et al., 2005). Allele frequencies and LD data from the 1000 Genomes Project Phase 3 were obtained through the 1000 Genomes Browser – Ensembl^2^, assembly GRCh37.p13, accessed on March 22, 2017. The list of htSNPs (*n* = 13) and their associated SNPs is provided in Supplementary Table 1. Genotyping of htSNPs was performed by Sequenom iPLEX matrix-assisted laser desorption/ionization time-of-flight mass spectrometry (Sequenom, San Diego, CA, United States) and using the Illumina BeadXpress platform (Illumina Inc., San Diego, CA, United States). Experimental design of PCR primers, extension primers and genotyping conditions were defined with the SpectroDESIGNER software (Sequenom).

### Statistical Analysis

Deviation from Hardy-Weinberg equilibrium was calculated for each htSNP using PLINK v1.07 (Purcell et al., 2007) and those deviating from the equilibrium (*P* < 0.05) were not retained for further analysis. OS was defined as the period of time between initiation of irinotecan treatment and death. PFS was defined as the period of time between initiation of irinotecan treatment and the first evidence of disease progression, death or last-follow-up. For response rate, patients with complete or partial response were compared to patients with stable disease or progression. Genetic associations with OS and PFS were tested using Cox proportional hazards model (SAS version 9.2, SAS Institute Inc., Cary, NC, United States). Associations with response rate and severe toxicities (neutropenia and diarrhea) were assessed by logistic regression. HRs and odds ratios (ORs) were adjusted for covariates including age, and co-treatment for the Canadian cohort and adjusted for gender, age, cancer primary site, stage at diagnosis, radical surgery and adjuvant chemotherapy in the Italian cohort, as performed in previous reports (Toffoli et al., 2006; Cecchin et al., 2009; Levesque et al., 2013; Chen et al., 2015a,b). Additive, dominant and recessive models were fitted independently. Each marker was tested independently in the two cohorts and then the results were compared. A marker was considered validated when the same effect using the same genetic model was observed in both populations, a trend observed in the Canadian cohort (*P* < 0.1) replicated in the Italian cohort with a *P*-value < 0.05. Analysis of pharmacokinetics parameters was performed using GraphPad Prism 5 (GraphPad Software Inc., La Jolla, CA, United States) and differences according to genotypes were tested using a two-tailed Mann–Whitney test. Gene expression data in tissues and associated genotypes were obtained from the GTEx Project portal (http://www.gtexportal.org/home/ on March 16, 2017). Analyses were carried out with GraphPad Prism 5 and differences among groups were tested using a two-tailed Mann–Whitney test. The datasets were obtained from dbGaP at http://www.ncbi.nlm.nih.gov/gap through dbGaP accession number phs000424.v6.p1; project ID 13346. Gene expression values were quantile normalized, as detailed on the GTEx portal. Median expression of genes relevant to irinotecan metabolism in RPKM (reads per kilobase per million mapped reads) from 119 normal liver samples was collected from the GTEx portal. Spearman’s correlation was used to assess correlation between gene expression.

### *In Vitro* Functional Assays

Sequences of primers used in this study are provided in Supplementary Table 4. The *HNF1A* cDNA was PCR amplified from a construct (FR_HNF1A #31104) kindly provided by Gerhart Ryffel (Addgene, Cambridge, MA, United States) (Senkel et al., 2005) that was subcloned into pcDNA3 *Eco*RI-*Eco*RV (Invitrogen, Carlsbad, CA, United States). This construct (pHNF1A_L27_N487) carried the minor alleles *rs1169288C* and *rs2264196A*, encoding respectively a leucine at position 27 and an asparagine at position 487. Mutagenesis was performed to obtain the major allele at position 487 (*rs2464196G*) and either the minor or the major allelle of rs1169288 (pHNF1A_L27_S487 or pHNF1A_I27_S487) using the Q5 Site-directed mutagenesis kit (New England Biolabs, Whitby, ON, Canada). A portion of the *ABCC1* promoter (-2.1 kb to -0.3 kb) was PCR amplified from genomic DNA and subcloned into pGL3-Basic vector (Promega Corp., Madison, WI, United States) between *SacI* and *XhoI* restriction sites upstream of the firefly luciferase gene. Sanger sequencing was used to verify the sequence of all constructs. HEK293 and HepG2 cells were obtained from ATCC (Rockville, MD, United States). Cell lines were grown as recommended and plated into 24-wells plates at a density of 1.0 × 10^5^ cells/well prior to transfection 24 h later. The pcDNA3_HNF1A constructs (200 ng) were co-transfected with the promoter/pGL3-Basic firefly luciferase reporter vector (200 ng) and pRL-CMV renilla luciferase vector as an internal control (20 ng, Promega Corp., Madison, WI, United States) using Lipofectamine 2000 (Invitrogen, Carlsbad, CA, United States) and OptiMEM-reduced serum media (Life Technologies, Burlington, ON, Canada) as per the manufacturer’s instructions. Cells were lysed 48h after transfection and assessed for luciferase activity using Dual-luciferase reporter assay kit (Promega Corp., Madison, WI, United States). Firefly luciferase activity is expressed as the ratio of firefly/renilla activities relatively to the corresponding control. Differences among conditions were tested by a Student’s two-tailed *t*-test.

## Results

Two cohorts comprising 167 mCRC Canadian patients and 250 mCRC Italian patients were studied, for a total of 417 mCRC patients all treated with the FOLFIRI regimen (leucovorin, 5-fluorouracil and irinotecan) (**Table 1**). Thirteen *HNF1A* htSNPs were assessed for their association with OS, PFS, response rate, severe gastrointestinal toxicities and severe neutropenia (listed in Supplementary Table 1). None of the tested *HNF1A* markers were associated with severe diarrhea in any of the two cohorts examined (data not shown). A few htSNPs were significantly associated with severe neutropenia in the Canadian cohort but not in the Italian cohort (Supplementary Table 2) whereas some htSNPs associated with OS and response rate were significant only in the Italian cohort (Supplementary Table 3).

**FIGURE 1:**
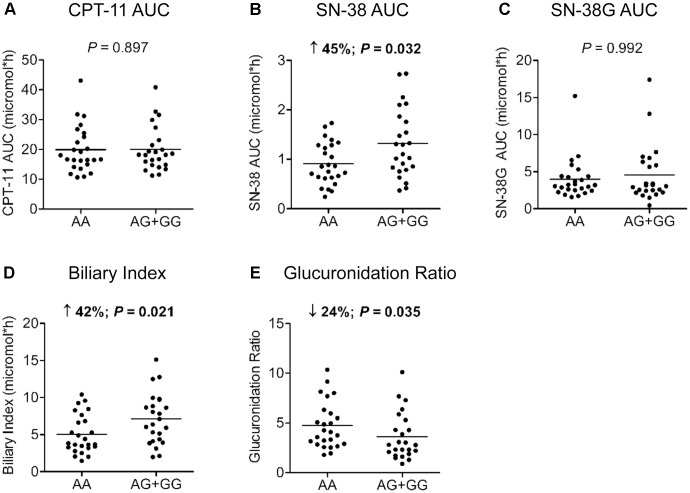
Association of *HNF1A* rs2244608A>G with pharmacokinetics parameters. Differences according to genotypes of 49 mCRC patients from the Italian cohort were tested for **(A)** irinotecan (CPT-11) AUC; **(B)** SN-38 AUC; **(C)** SN-38 glucuronide (SN-38G) AUC; **(D)** biliary index and **(E)** glucuronidation ratio using a two-tailed Mann–Whitney test. The bars indicate mean values for each group. Biliary index was calculated with the formula: [CPT-11 AUC X (SN-38 AUC/SN-38G AUC)] and glucuronidation ratio with the formula: (SN-38G AUC/SN-38 AUC). AUC, area under the curve.

For *HNF1A* intronic variant rs2244608A>G, a trend for an improved PFS in the Canadian population with a HR of 0.74 (95% CI = 0.53 – 1.03, *P* = 0.076) was observed. The same effect using the same genetic model was significant in the Italian cohort with a HR of 0.72 (95% CI = 0.53 – 0.98, *P* = 0.038) (**Table 2**). A strong association between rs2244608A>G and improved PFS was also found when both cohorts were combined and analyzed with the same genetic model, adjusted for age and co-treatment, with a HR of 0.72 (95% CI = 0.59 – 0.89, *P* = 0.002). Consistent with a potential significant influence of this genetic variation, in the analysis of its association with the pharmacokinetics parameters of irinotecan in a subset of patients of the prospective Italian cohort, carriers of the *rs2244608G* allele (*n* = 24) presented an enhanced blood exposure to the active metabolite SN-38 (increase of 45% of SN-38 AUC, *P* = 0.032) compared to carriers of homozygous rs2244608AA genotype (*n* = 25) whereas no significant changes were noted for CPT-11 AUC and SN-38G AUC (**Figures 1A–C**). Carriers of the *rs2244608G* allele also had a 41% increased biliary index (*P* = 0.021) and a 24% decreased glucuronidation ratio (*P* = 0.035) compared to homozygote carriers of *rs2244608A* allele (**Figures 1D,E**).

**Table 2:** Association between *HNF1A* polymorphisms and progression-free survival (PFS).

		Canadian cohort	Italian cohort
			
htSNPs^a^	Model	HR^b^ (95% CI); *P*-value	HR^c^ (95% CI); *P*-value
rs2244608A>G	Dominant	0.74 (0.53 – 1.03); 0.076	0.72 (0.53 – 0.98); 0.038
rs1169286T>C	Dominant	0.66 (0.46 – 0.95); 0.024	Non-significant
rs2393791T>C	Dominant	0.69 (0.50 – 0.97); 0.032	Non-significant
rs12427353G>C	Dominant	1.36 (0.97 – 1.90); 0.074	Non-significant
rs2071190T>A	Additive	Non-significant	1.35 (1.04 – 1.74); 0.022
rs1169302T>G	Recessive	Non-significant	1.54 (1.03 – 2.31); 0.038
rs2259820C>T	Dominant	0.69 (0.50 – 0.96); 0.026	Non-significant
rs1169307C>T	Recessive	1.57 (1.02 – 2.40); 0.040	Non-significant
rs735396T>C	Dominant	Not in HWE	0.72 (0.54 – 0.97); 0.033


***^a^**Additional genotyped htSNPs were not significantly associated with PFS (rs1882149, rs3999413, rs1169303, rs1169293) or were already tagged (*r*^*2*^ ≥ 0.98) by another htSNP presented here. ^b^Adjusted for age and co-treatment. ^c^Adjusted for gender, age, cancer site, stage at diagnosis, radical surgery and adjuvant chemotherapy. Base changes are given for the + strand. 95% CI, 95% confidence interval; HR, hazard ratio; htSNP, haplotype-tagging single nucleotide polymorphism; HWE, Hardy-Weinberg equilibrium.*

**FIGURE 2:**
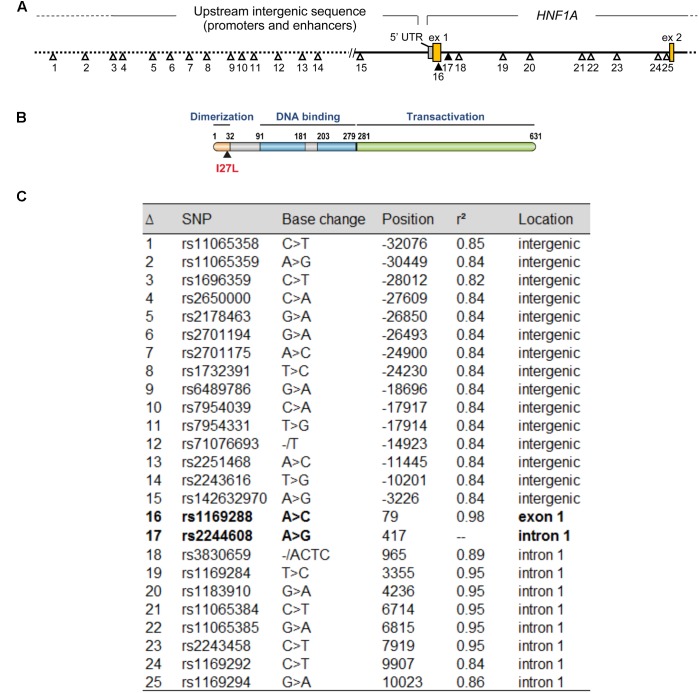
The *HNF1A* marker rs2244608A>G associated with improved PFS is tightly linked (*r*^2^ = 0.98) to a number of other SNPs in the *HNF1A* gene including the non-synonymous coding variant rs1169288A>C (p.I27L). **(A)** Genetic variants in strong linkage disequilibrium (LD) (*r*^2^ > 0.80) with the intronic marker rs2244608 significantly associated with outcome in both populations of mCRC patients treated with irinotecan are represented on the *HNF1A* gene locus. The positive intronic marker rs2244608 and the non-synonymous rs1169288 variants, represented in bold, are tightly linked (*r*^2^ = 0.98) in individuals of European descent. **(B)** Representation of the HNF1A protein and coordinates in amino acids of its main protein domains. **(C)** Position (relative to the translation start site) and LD values are shown for polymorphisms presented in A. LD values are from the 1000 Genomes Phase 3 CEU population (European descent).

**FIGURE 3:**
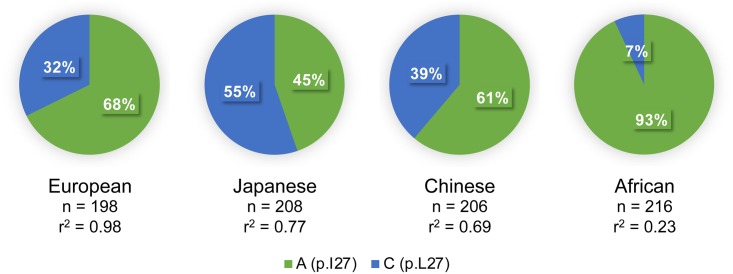
Frequency of the non-synonymous rs1169288A>C variation in diverse ethnic groups and its linkage with the intronic marker rs2244608A>G associated with outcome. Linkage disequilibrium (*r*^2^) is given for each population. Data were obtained from the 1000 Genomes Phase 3 project. *n*, number of genotyped alleles. The rs2244608A>G marker is associated with outcome in mCRC patients of European descent.

The minor allele frequency of rs2244608A>G is 0.32 in the CEU population, 0.34 in the Canadian cohort and 0.33 in the Italian cohort. The htSNP rs2244608A>G is located in the first intron of *HNF1A* and is in strong LD with 24 other SNPs in individuals of European descent (*r*^2^ > 0.80 from 1000 Genomes Phase 3 CEU population) (**Figure 2**), namely with the non-synonymous coding variant rs1169288c.79A>C located in the first exon of *HNF1A*, with *r*^2^ of 0.98. The minor allele *rs1169288C* introduces the substitution of the isoleucine 27 to a leucine (p.I27L) in the dimerization domain of HNF1A (**Figure 2B**) and therefore could functionally contribute to changes observed in circulating SN-38 levels for carriers of the minor *rs2244608G* allele in almost complete linkage with the minor *rs1169288C* (p.27L) allele. However, the degree of linkage between the positive marker rs2244608A>G and the coding variant rs1169288A>C varies significantly across ethnic groups. The strongest linkage was observed in individuals of European descent (*r*^2^ = 0.98), followed by Asians (Japanese *r*^2^ = 0.77 and Chinese *r*^2^ = 0.69) and Africans (*r*^2^ = 0.23) (**Figure 3**). All other linked polymorphisms are intronic or located in the untranscribed region upstream of the *HNF1A* gene, and may influence the expression of *HNF1A*.

**FIGURE 4:**
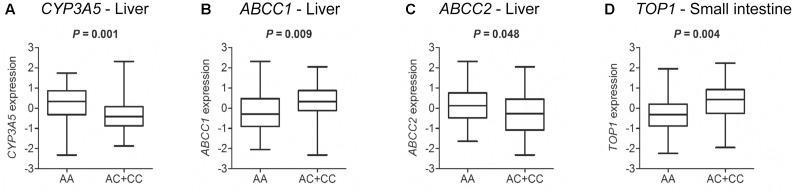
The *HNF1A rs1169288C* variant allele (p.L27) is associated with altered expression of *CYP3A5*, *ABCC1* and *ABCC2* transporter genes, and irinotecan drug target *TOP1*. Gene expression and genotypes were from individuals of the GTEx cohort. *P*-values were calculated by a two-tailed Mann–Whitney test. **(A–C)** Normal livers (*n* = 89 individuals): rs1169288AA, *n* = 51; rs1169288AC/CC, *n* = 38; **(D)** Normal small intestines (*n* = 75 individuals): rs1169288AA, *n* = 42; rs1169288AC/CC, *n* = 33.

**FIGURE 5:**
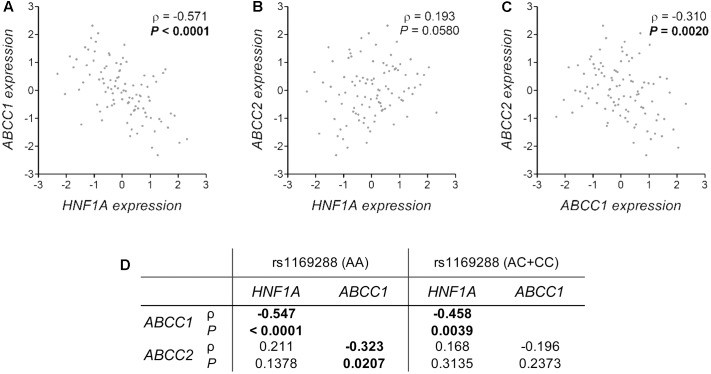
Correlation of the hepatic expression of *HNF1A*, *ABCC1* and *ABCC2* and the influence of the rs1169288 genotype. **(A–C)** Correlations are shown for 97 individuals of the GTEx cohort. **(D)** Spearman correlation ρ coefficients and *P*-values for genotyped individuals carrying rs1169288AA (homozygous for p.I27; *n* = 51) and rs1169288AC/CC (heterozygous and homozygous p.L27; *n* = 38) genotypes.

The analysis of liver tissues from 119 healthy donors indicated that *HNF1A* as well as *ABCC1* and *ABCC2* are expressed (data not shown). In support of a potential functional significance of the coding *HNF1A* variant rs1169288 and based on the analysis of liver samples from 89 healthy donors with available genotype data, we observed an enhanced hepatic expression of *ABCC1* (*P* = 0.009) and a lower expression of *ABCC2* (*P* = 0.048) and *CYP3A5* (*P* = 0.001) in carriers of the minor *rs1169288C* allele (*n* = 38 individuals) compared to those carrying the rs1169288AA genotype (*n* = 51 individuals) (**Figures 4A–C**). In addition, compared to homozygous carriers of *rs1169288A* allele (*n* = 42 individuals), expression of the irinotecan drug target gene *TOP1* was significantly higher in the small intestine (*P* = 0.004) in carriers of the minor allele *rs1169288C* (*n* = 33 individuals) (**Figure 4D**). We also noted that the hepatic expression of *HNF1A* is inversely correlated to *ABCC1* (Spearman ρ = -0.571, *P* < 0.0001) and a trend for a positive correlation with *ABCC2* (Spearman ρ = 0.193, *P* = 0.058) was observed (**Figures 5A,B**). In line, a negative correlation between *ABCC1* and *ABCC2* expressions was observed (Spearman ρ = -0.310, *P* = 0.002, **Figure 5C**). When accounting for the rs1169288 genotype, the negative correlation between expression of *ABCC1* and *HNF1A* remained significant in both genotype groups. The negative correlation between *ABCC1* and *ABCC2* was also preserved but was significant only in homozygous carriers of the reference *rs1169288A* allele (n = 51), likely due the smaller sample size for *rs1169288C* carriers (*n* = 38) (**Figure 5D**). Relationship to other pathways relevant to irinotecan is provided in Supplementary Figure 1, but none reached significance. A trend was observed for carriers of *rs1169288C* allele and a decreased *HNF1A* expression (*P* = 0.082).

**FIGURE 6:**
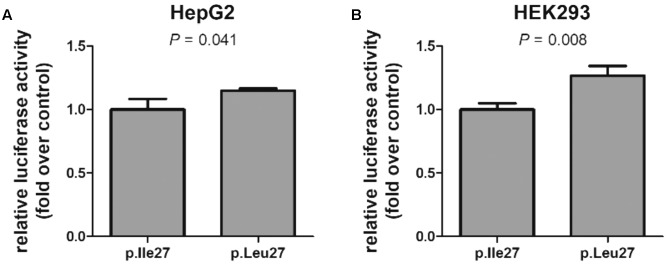
Functional impact of the *HNF1A* rs1169288 coding variant. Transcriptional induction of the *ABCC1* promoter by HNF1A p.I27 (p.Ile27) and p.L27 (p.Leu27) in liver **(A)** and kidney **(B)** cells. Cells were co-transfected with a reporter vector containing the firefly luciferase gene downstream of the *ABCC1* promoter and an *HNF1A* construct (p.Ile27 or p.Leu27). The bars represent the mean ± SD of three independent experiments performed in triplicate. Statistical analysis used Student’s two-tailed *t*-test.

Based on the prominent association between HNF1A and ABCC1 observed in human liver samples, we then tested whether the p.I27L substitution affects the ability of transcription factor HNF1A to induce transcription of a reporter gene construct containing the *ABCC1* promoter. *In vitro* luciferase assays revealed a significant impact of the p.I27L substitution when tested in the human HEK293 kidney cells and the liver HepG2 cell line with a significant increased in reporter gene activity associated with HNF1A p.L27 by 27% (*P* = 0.008) and 15% (*P* = 0.041), respectively, compared to cells expressing the HNF1A p.I27 protein (**Figure 6**).

## Discussion

We report that the intronic htSNP rs2244608A>G located in the *HNF1A* gene may be predictive of an improved PFS in two independent cohorts of 417 mCRC white patients treated with irinotecan-based regimen. In support of a better response to chemotherapy, an enhanced systemic exposure to the active metabolite of irinotecan, SN-38, was observed in a subset of mCRC patients carrying the minor *rs2244608G* allele associated with prolonged PFS. The rs2244608A>G was not associated with severe toxicities in the Canadian and the Italian cohorts, suggesting that the increase in SN-38 exposure for carriers of *rs2244608G* allele may not induce severe side effects. In individuals of European descent, rs2244608A>G marker is almost in complete linkage (*r*^2^ = 0.98) with the non-synonymous coding variant rs1169288A>C (p.I27L) located in exon 1, which could explain the observed phenotype. This is reinforced by an altered hepatic expression of metabolic and transport genes involved in irinotecan pharmacology, in favor of superior blood levels of the anticancer drug coupled with enhanced expression of irinotecan target gene *TOP1* in the intestine, in carriers of the *rs1169288C* allele strongly linked to the *rs2244608G* marker. In functional *in vitro* studies, the HNF1A *rs1169288C* p.L27 polymorphism could significantly increase the *ABCC1* promoter activity in human HepG2 hepatocellular carcinoma cell line and HEK293 kidney cells. This is the first study to demonstrate the relationship between variability in the transcription factor *HNF1A* gene and clinical outcome in the context of irinotecan-based regimen.

HNF1A plays an important part in the pathogenesis of various diseases particularly diabetes and cardiovascular diseases. Genetic variations in the *HNF1A* gene, and especially the coding variant rs1169288A>C (p.I27L), have been previously linked to type 2 diabetes (T2D) (Holmkvist et al., 2006; Gaulton et al., 2015), gestational diabetes mellitus (Shaat et al., 2006), cholelithiasis (Richter et al., 2012), coronary artery diseases (CAD) (Wang et al., 2015; Zhou et al., 2017), serum lipid levels (Babaya et al., 2003; Zhou et al., 2017) and levels of the biomarker of inflammation C-reactive protein (CRP) (Reiner et al., 2008; Reiner et al., 2009), suggesting a functional impact of the codon 27 amino acid substitution. However, this does not preclude the potential impact of the intronic rs2244608 marker (or other SNPs in high LD), which can influence gene expression, stability or splicing of the mRNA. The p.I27L substitution is located in the N-terminal dimerization domain of HNF1A that mediates its homodimerization and heterodimerization with HNF1B and the dimerization cofactor DcoH (Narayana et al., 2001). This conservative substitution may be anticipated to have little impact on the structure of HNF1A; however, isoleucine 27 is highly conserved across species, supporting a functional significance (Narayana et al., 2001; Setoh et al., 2015).

**FIGURE 7:**
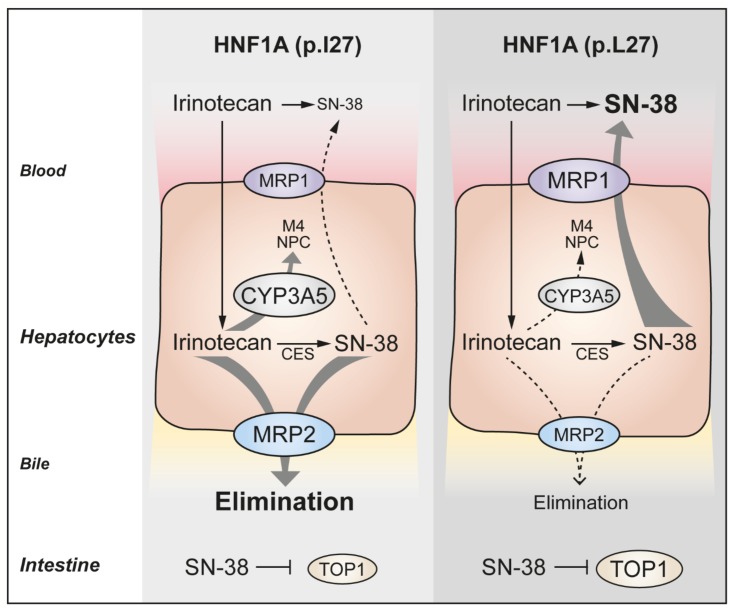
Proposed model for the underlying mechanism of an improved progression-free survival associated with the HNF1A *rs1169288C* allele.

Of key genes involved in irinotecan pharmacology, the hepatic expression of *CYP3A5* and the transporters *ABCC1* and *ABCC2* was significantly influenced by the rs1169288A>C. The expression of these genes may be regulated by HNF1A, either directly or indirectly. In line, the expression of *ABCC2* is regulated by HNF1A in liver cells (Odom et al., 2004; Qadri et al., 2009). In addition, results of our *in vitro* investigations allowed to further substantiate evidence for a functional impact of variant HNF1A p.I27L (rs1169288A>C) on the expression of *ABCC1* in human liver and kidney cells, with an increased transcriptional induction by HNF1A p.L27 using the *ABCC1* promoter. Consistent with the elevated SN-38 AUC, higher *ABCC1* (MRP1) expression and lower *ABCC2* (MRP2) and *CYP3A5* expressions were observed for *rs224608G* carriers, strongly linked to the *rs1169288C* allele. MRP1 is on the basolateral membrane of hepatocytes (Emi et al., 2013) and mediates efflux of SN-38 into blood (Chen et al., 1999) whereas MRP2 is located on the apical membrane of hepatocytes (Emi et al., 2012) and is involved in the efflux of several endogenous compounds and drugs, including irinotecan and its metabolites, to the bile (Chu et al., 1997; Sugiyama et al., 1998). The superior exposure to SN-38 in carriers of *rs2244608G* may thus be explained through an enhanced redirection of SN-38 from hepatocytes to blood circulation and a lower excretion of irinotecan metabolites in the bile. In line with this hypothesis, Wang et al. (2016) demonstrated that hepatic inhibition of MRP2 expression with hesperidin in rats lead to an increased SN-38 exposure while biliary excretion of SN-38 was reduced. Furthermore, the rs1169288 polymorphism has been linked to altered irinotecan pharmacokinetics in a study of 85 patients with solid tumors (Rosner et al., 2008). The strong negative correlation between hepatic expression of *ABCC1* and *HNF1A* and the nearly significant positive correlation between *ABCC2* and *HNF1A* observed in the GTEx cohort sustain a functional interaction, yet an inverse regulatory influence of HNF1A on these two transporters. We could thus speculate that a decreased expression or function of *HNF1A* would lead to increase *ABCC1* expression and potentially decreased *ABCC2* expression. This might contribute to an enhancement of SN-38 systemic exposure and a reduced elimination of the drug, leading to a prolonged PFS, as represented in **Figure 7**. Consistent with this notion, Holmkvist et al. (2006) demonstrated a decreased transcriptional induction of *SLC2A2* promoter (Solute Carrier Family 2 Member 2 or GLUT2) by HNF1A p.L27 in HeLa cells. Although there are inconsistencies in the literature, previous reports also support a link between genetic variations in the *ABCC1* and *ABCC2* genes, altered irinotecan pharmacokinetics and clinical outcomes in colon and lung cancer patients (Rosner et al., 2008; Innocenti et al., 2009; Akiyama et al., 2012; Li et al., 2016).

The impact of variations in HNF1A expression and/or function is likely to be multifactorial and may affect multiple pathways relevant to irinotecan pharmacokinetics and pharmacodynamics (Smith et al., 2006). In the liver of healthy individuals, we further found a positive correlation between *HNF1A* and *CYP3A5* expressions, in line with a previous observation by Noll et al. (2016), and an association with reduced expression of *CYP3A5* for the *rs1169288C* allele. As CYP3A5 oxidizes irinotecan in inactive metabolites, reduction of its expression may increase the proportion of irinotecan available for conversion into SN-38 by carboxylesterases, resulting in higher SN-38 exposure. Lastly, carriers of *rs1169288C* also presented higher expression of the irinotecan drug target gene *TOP1* in the small intestine, which could explain, at least in part, a better response to TOP1 inhibitor treatment and improved PFS (**Figure 7**). Additional functional studies will be required to test these hypotheses.

The strengths of our study include the examination of two independent cohorts of mCRC comprising 417 white patients and the analysis of pharmacokinetics and transcriptomic data to further investigate the potential mechanisms underlying the association. Given its exploratory nature, we did not apply correction for multiple testing. Besides, the study design involved an independent set of patients used to replicate findings observed in the training set, which reduces the likelihood of false-positive results while maximizing the detection of true-positive associations. Whereas this may result in potential false-positive associations, the high stringency of multiple testing may have led to false-negative associations due to the number of patients included. The LD between rs2244608A>G and rs1169288A>C was almost complete in individuals of European descent but is lower in African and Asian populations implying that our findings may be most particularly significant in individuals of European descent. While recognizing the limitations of our study, further investigations are warranted to replicate our findings and substantiate the functional impact of the HNF1A p.I27L coding variation on pathways relevant to drug metabolism, transport and action.

## Conclusion

We report the identification of rs2244608A>G, strongly linked to the functional coding variant *HNF1A* rs1169288A>C (p.I27L), as a predictive marker of improved PFS in mCRC patients treated with irinotecan-based chemotherapy. Prolonged PFS may likely be explained by a greater exposure to SN-38, possibly due to an altered transcriptional regulation by HNF1A of genes involved in irinotecan pharmacology including *CYP3A5*, *ABCC1* and *ABCC2* transporters and *TOP1*.

## Author Contributions

Conceptualization: CG and GT; Methodology: All authors; Investigation: AL, EDM, EC, EL, DJ, FC, AB, MD, LV, and CG; Formal Analysis: AL, EDM, EC, EL, LV, GT, and CG; Writing – Review and Editing: All authors; Supervision: CG and GT; Funding acquisition: CG and GT.

## Conflict of Interest Statement

The authors declare that the research was conducted in the absence of any commercial or financial relationships that could be construed as a potential conflict of interest.

## Footnotes

1 http://www.hapmap.org

2 http://grch37.ensembl.org/index.html

## Abbreviations

AUC: area under the curve
CEU population: Utah residents with Northern and Western European ancestry population
FOLFIRI: folinic acid (leucovorin) + 5-fluorouracil (5-FU) + irinotecan
HR: hazard ratio
htSNP: haplotype-tagging single nucleotide polymorphism
LD: linkage disequilibrium
mCRC: metastatic colorectal cancer
OR: odds ratio
OS: overall survival
PFS: progression-free survival
