# Supplementary material for: Improved Progression-Free Survival in Irinotecan-Treated Metastatic Colorectal Cancer Patients Carrying the HNF1A Coding Variant p.I27L
Source: Front Pharmacol. 2017 Oct 10;8:712. doi: 10.3389/fphar.2017.00712 (PMC5641335; doi:10.3389/fphar.2017.00712)
Supplement: Supplementary file 1 [file Table_1.pdf]

Improved progression-free survival in irinotecan-treated metastatic colorectal cancer patients carrying the HNF1A coding variant p.I27L

Adrien Labriet[1,2†], Elena De Mattia[3†], Erika Cecchin[3], Éric Lévesque[4,5], Derek Jonker[6], Félix Couture[4,5], Angela Buonadonna[7], Mario D'Andrea[8], Lyne Villeneuve[1,2], Giuseppe Toffoli[3] and Chantal Guillemette[1,2*]

*Corresponding Author: Chantal Guillemette, Ph.D., CHU de Québec Research Center, R4701.5, 2705 Blvd. Laurier, Québec, Canada, G1V 4G2. E-mail: chantal.guillemette@crchudequebec.ulaval.ca

Supplementary Tables

Supplementary Tables

Supplementary Table 1. *HNF1A* haplotype-tagging single nucleotide polymorphisms (htSNPs) in moderate to weak linkage disequilibrium ($r^2 < 0.8$), and their corresponding associated SNPs ($r^2 > 0.8$) as determined with the data of 1000 Genomes Phase 3 for the CEU population (European descent).

htSNPs	Associated SNPs				
rs2244608	rs1169288	rs2243458	rs142632970	rs7954039	rs2178463
	rs1169284	rs3830659	rs2243616	rs6489786	rs2650000
	rs1183910	rs1169294	rs2251468	rs1732391	rs11065359
	rs11065384	rs11065358	rs71076693	rs2701175	rs1696359
	rs11065385	rs1169292	rs7954331	rs2701194	
rs1169286	-				
rs2393791	rs9738226	rs7979478	rs7139079		
	rs2393776	rs7979473	rs7135337		
	rs7970695	rs7310409	rs7953249		
	rs2393775	rs1169289	rs11065365		
	rs546442868	rs2464190			
rs12427353	rs34565150	rs67075643	rs71453526		
	rs148285095	rs12425790	rs112508908		
	rs5801412	rs7131696	rs7965349		
	rs55834942	rs7957197			
	rs55879344	rs61953352			
rs2071190	-				
rs1169302	rs1169299				
rs2259820	rs2464196	rs2257764	rs2264778		
	rs1169301	rs2264750	rs2258287		
	rs1169300	rs1182933	rs1169281		
	rs1169314	rs3213545	rs1169279		
	rs1169315	rs1169291			
rs1169307	rs2257813				
	rs2264779				
rs735396	rs1169309	rs2259816	rs2257962		
	rs1169306	rs2464195	rs2254971		

	rs1169310	rs2259852	rs4278584	
	rs1169311	rs34521980		
	rs1169312	rs2264782		
rs1882149	rs41279096	rs2393777	rs73228105	rs7303401
	rs56383076	rs201044504	rs7961178	rs7132164
	rs2393774	rs73228108	rs2251556	rs73226290
	rs55691424	rs55956549	rs73226296	rs73226268
	rs73214155	rs2254779	rs7135458	
rs3999413	rs1169304	rs1169280	rs12811390	
	rs3999412	rs2259697		
	rs10849828	rs12819210		
	rs11065393	rs535217845		
	rs2258227	rs11065403		
rs1169303	rs2258043			
	rs2701180			
rs1169293	rs2245407	rs1971317	rs12313339	
	rs113154140	rs73226270	rs113277649	
	rs959400	rs1696360	rs2708086	
	rs73226274	rs570954040		
	rs9919745	rs1617471		

Abbreviations: htSNP, haplotype-tagging SNP; SNP, single nucleotide polymorphism.
Associated SNPs are polymorphisms in strong linkage with the htSNP with $r^2 > 0.8$, as determined with the data of 1000Genomes Phase 3 for the CEU population.

Supplementary Table 2. Association between *HNF1A* htSNPs and severe neutropenia in the Canadian cohort. None of the htSNPs were significant in the Italian cohort using the same genetic model.

**htSNPs**	**Model**	**OR[a] (95% CI); *P*-value**
rs1169286T>C	Dominant	0.45 (0.19 - 1.07); 0.070
rs2393791T>C	Dominant	0.45 (0.20 - 1.04); 0.061
rs12427353G>C	Recessive	8.58 (1.30 - 56.57); 0.025
rs2071190T>A	Recessive	2.70 (0.85 – 8.51); 0.091
rs1169302T>G	Dominant	0.48 (0.21 - 1.11); 0.085
rs2259820C>T	Dominant	0.32 (0.13 - 0.78); 0.012
rs1169303A>C	Dominant	0.35 (0.15 - 0.84); 0.018
rs1169307C>T	Recessive	3.38 (1.31 - 8.73); 0.012

[a]Adjusted for age and co-treatment. Base changes are given for the + strand. Abbreviations: 95% CI, 95% confidence interval; OR, odds ratio; htSNP, haplotype-tagging single nucleotide polymorphism.

Supplementary Table 3. Association between *HNF1A* htSNPs and overall survival and response rate in the Italian cohort. None of the htSNPs were significant in the Canadian cohort for these endpoints using the same genetic model.

**Overall survival**	**Model**	**HR[a] (95% CI); P-value**
rs2393791T>C	Dominant	0.61 (0.42 – 0.88); 0.008
rs1169302T>G	Recessive	1.78 (1.13 – 2.79); 0.013
rs1169307C>T	Recessive	1.69 (1.07 – 2.67); 0.026
**Response rate**	**Model**	**OR[a] (95% CI); P-value**
rs12427353G>C	Dominant	0.20 (0.05 – 0.78); 0.020

[a]Adjusted for gender, age, cancer site, stage at diagnosis, radical surgery and adjuvant chemotherapy. Base changes are given for the + strand. Abbreviations: 95% CI, 95% confidence interval; HR, hazard ratio; htSNP, haplotype-tagging single nucleotide polymorphism; OR, odds ratio.

Supplementary Table 4. Sequences of primers used for *in vitro* studies.

**Primers**	**Sequence (5' → 3')**
HNF1A-EcoRI-forward (PCR and cloning of *HNF1A* ORF)	CTAGCTGGAATTCCCATGGAGCAAAAGCTCATT
HNF1A-EcoRV-reverse (PCR and cloning of *HNF1A* ORF)	CTAGCTGGATATCCCCAAGCAGGCAGTACAGG
rs1169288A-forward (site directed mutagenesis)	AGAGGCACTGaTCCAGGCACT
rs1169288A-reverse (site directed mutagenesis)	TTGCTCAGCCCTGACTCC
rs2464196G-forward (site directed mutagenesis)	GTGACCCAGAgCCCCTTCATG
rs2464196G-reverse (site directed mutagenesis)	ATGGCTCTGCACAGGTGG
prom*ABCC1*-Sac-forward (PCR and cloning of *ABCC1* promoter)	CTAGCTGGAGCTCGCACTGAGATTACATGCGTGA
prom*ABCC1*-Xho1-reverse (PCR and cloning of *ABCC1* promoter)	CTAGCTGCTCGAGCCCAGATCCTCCAAGGCTTA
