# Supplementary figures and images for: Improved Progression-Free Survival in Irinotecan-Treated Metastatic Colorectal Cancer Patients Carrying the HNF1A Coding Variant p.I27L
Source: Front Pharmacol. 2017 Oct 10;8:712. doi: 10.3389/fphar.2017.00712 (PMC5641335; doi:10.3389/fphar.2017.00712)

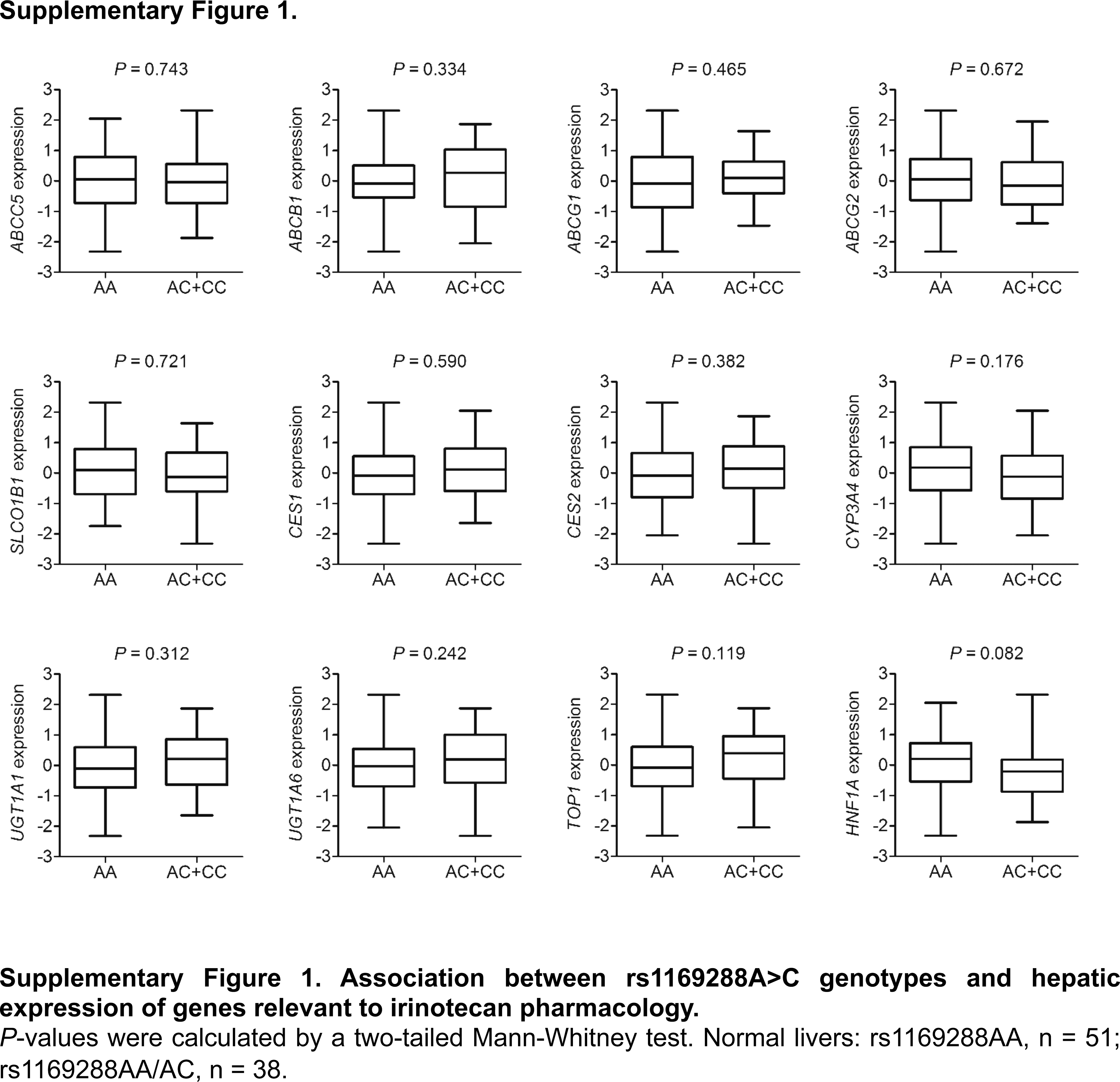

Supplement: Supplementary file 2 [file Image_1.TIF]
